# Rapid DNA analysis for automated processing and interpretation of low DNA content samples

**DOI:** 10.1186/s13323-016-0033-7

**Published:** 2016-03-17

**Authors:** Rosemary S. Turingan, Sameer Vasantgadkar, Luke Palombo, Catherine Hogan, Hua Jiang, Eugene Tan, Richard F. Selden

**Affiliations:** NetBio, 266 Second Avenue, Waltham, MA 02451 USA

**Keywords:** Rapid DNA analysis, Low DNA content samples, Ultrafiltration, STR profiles, CODIS

## Abstract

**Background:**

Short tandem repeat (STR) analysis of casework samples with low DNA content include those resulting from the transfer of epithelial cells from the skin to an object (e.g., cells on a water bottle, or brim of a cap), blood spatter stains, and small bone and tissue fragments. Low DNA content (LDC) samples are important in a wide range of settings, including disaster response teams to assist in victim identification and family reunification, military operations to identify friend or foe, criminal forensics to identify suspects and exonerate the innocent, and medical examiner and coroner offices to identify missing persons. Processing LDC samples requires experienced laboratory personnel, isolated workstations, and sophisticated equipment, requires transport time, and involves complex procedures. We present a rapid DNA analysis system designed specifically to generate STR profiles from LDC samples in field-forward settings by non-technical operators. By performing STR in the field, close to the site of collection, rapid DNA analysis has the potential to increase throughput and to provide actionable information in real time.

**Results:**

A Low DNA Content BioChipSet (LDC BCS) was developed and manufactured by injection molding. It was designed to function in the fully integrated Accelerated Nuclear DNA Equipment (ANDE) instrument previously designed for analysis of buccal swab and other high DNA content samples (Investigative Genet. 4(1):1–15, 2013). The LDC BCS performs efficient DNA purification followed by microfluidic ultrafiltration of the purified DNA, maximizing the quantity of DNA available for subsequent amplification and electrophoretic separation and detection of amplified fragments. The system demonstrates accuracy, precision, resolution, signal strength, and peak height ratios appropriate for casework analysis.

**Conclusions:**

The LDC rapid DNA analysis system is effective for the generation of STR profiles from a wide range of sample types. The technology broadens the range of sample types that can be processed and minimizes the time between sample collection, sample processing and analysis, and generation of actionable intelligence. The fully integrated Expert System is capable of interpreting a wide range or sample types and input DNA quantities, allowing samples to be processed and interpreted without a technical operator.

## Background

Military, law enforcement, and intelligence investigations are often focused on collecting forensic evidence left behind by persons involved in terrorist, criminal, or other illegal activities. The biological evidence recovered includes epithelial cells that are transferred by casual handling of objects, colloquially referred to as “touch DNA samples” [1–5]. Touch samples include fingerprints, skin cells found on firearms and clothing (e.g., a shirt collar), and oral epithelial cells found on the opening of a soda can or the rim of a drinking glass. The quantity of DNA that is recovered from touch DNA samples is highly variable, ranging from less than 6 pg—the quantity of genomic DNA in a single human cell—to 100 ng. Small blood spatter stains and minute tissue fragments may also contain less than 100 ng of DNA and, taken together with touch samples, may be referred to generally as “low DNA content (LDC)” samples.

Considerable efforts have been made to understand and improve methodologies associated with LDC samples, and a range of techniques have been developed to enhance process steps from sample collection through electrophoretic detection of short tandem repeat (STR) fragments. Improvements in sample collection include optimized swabbing of the target area [6–8], the use of alternative moistening agents to enhance retrieval [9–12], and optimal swab matrix selection [9, 13–15]. With respect to DNA purification, silica-based DNA purification methods are generally preferred over conventional Chelex and organic extraction methods to minimize sample loss during the process [16–21]. Improvements in primers used in the core STR systems and novel multiplex polymerase chain reaction (PCR) assays [22, 23], increasing the number of PCR cycles [24, 25], and post-PCR concentration and clean-up steps [26–29] have also assisted typing of LDC samples. Nonetheless, despite all these significant advances, processing LDC samples in the conventional laboratory is still laborious and time-consuming.

We have previously developed the Accelerated Nuclear DNA Equipment (ANDE) Rapid DNA Analysis system, a field-deployable, rapid, and fully integrated DNA analysis system that can automatically generate and interpret STR profiles from buccal swabs in 84 min [30]. The technology significantly decreases processing time from sample collection to generation of STR profiles, and the instrument incorporates features to allow for use outside the lab including ruggedization for transport, operability in environmental extremes, room temperature reagent stability, data security, and ease of use. The system is undergoing developmental validation testing for National DNA Index System (NDIS) approval. Swab samples are inserted into the swab chamber of the disposable single-use BioChipSet (BCS), and an integrated Expert System analyzes and presents the resulting profiles. One important property of the ANDE system is the flexibility of its platform. The modular design of the DNA purification, STR amplification, and microfluidic electrophoresis regions of the BCS allows a wide range of sample types and assays to be analyzed in a rapid DNA format.

Here, we present a modification of the ANDE system to allow rapid analysis of LDC samples. The BCS consumable has been modified for samples with LDC, but the core elements and dimensions of the consumable, instrument, sample tracking, and system operation have been maintained. An important feature of the LDC BioChipSet is the incorporation of a microfluidic ultrafiltration module. This module enables sample concentration following DNA purification to maximize capture of DNA for subsequent processing steps. The LDC Rapid DNA Analysis system offers significant improvement in the sensitivity and limit of detection. The incorporation of the fully integrated Expert System is critical in that it enables interpretation of a wide range or sample types and input DNA quantities, allowing samples to be processed and interpreted without a technical operator. Taken together, the system offers the potential to accelerate human identification in criminal forensic, medical examiner, disaster victim identification and family reunification, and military applications.

## Methods

### Low DNA content BioChipSet design and fabrication

**Fig. 1:**
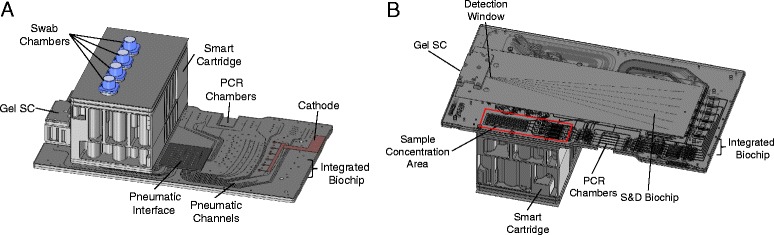
The Low DNA Content (LDC) BioChipSet cassette. **a** Schematic diagram, *top view*. The Smart Cartridge accepts four LDC samples and contains the liquid reagents required for sample processing. Fluids are transported throughout the biochip by pneumatic pressure—there are no moving parts. **b** *Bottom view*. The electrophoretic separation region, laser detection window, and sample concentration modules are shown

The LDC biochipset (Fig. 1) is injection-molded using cyclic olefin polymer and is a single-use, disposable device with all reagents preloaded. The biochipset accepts four samples; all reagents have been shown to be room temperature stable for at least 6 months [30]. All instruments to biochipset interfaces (e.g., pneumatic, thermal, electrical) in the LDC biochipset are identical to those of the High DNA Content (HDC) biochipset [31] in order to allow both consumables to function using the identical instrument. The ANDE instrument automatically detects the BCS type using an RFID reader and selects the required sample processing protocol.

### Sample concentration by UF

**Fig. 2:**
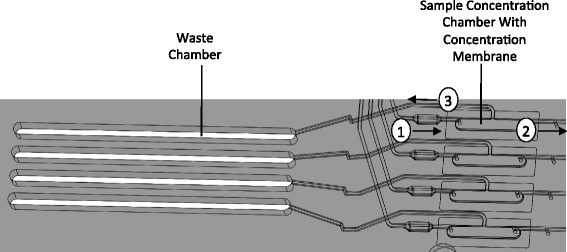
Sample concentration module. Schematic diagram of the four concentration channels, one for each independent sample. Purified DNA solution flows into the concentration chamber (*arrow 1*) from the purification module. Concentrated DNA solution in the retenate flows out of the concentration chamber (*arrow 2*) to the PCR chamber. Permeate is collected in the waste chamber (*arrow 3*)

A sample concentration module was incorporated into the LDC BioChipSet by positioning it downstream of the purification module (Fig. 1). Figure 2 is an expanded view of this module. The purified DNA solution is directed to the semipermeable UF membrane by application of pneumatic pressure. The membrane prevents large genomic DNA fragments from flowing through but allows elution solution to flow through the membrane pores into a waste chamber. As a result, DNA present in the retentate is concentrated, with the degree of concentration determined by the efficiency of DNA recovery and the final volume of the retentate. The DNA concentration membrane was selected based on its ability to efficiently retain DNA molecules, meet desired flow rates, and exhibit chemical compatibility with the BCS fabrication processes. Figure 1b shows the location of the sample concentration module on the LDC BCS, and Fig. 2 shows an expanded view of the module. Purified DNA directed is through the UF membrane by application of pneumatic pressure.

### Sample collection

Sample collections were performed using SecurSwab™ DNA collector (http://www.bodetech.com/PDF/SecurSwab_instructions.pdf) modified with a locking plastic cap containing an RFID chip for sample tracking. A variety of additional commercial swab types, including flocked, foam, and cotton, have been utilized by introducing the cut swab head into the sample chamber. These swab types function equivalently to the modified SecurSwab but lack the sample tracking feature. The study did not require Institutional Review Board approval, and participants consented to provide non-clinical samples for the study.

- *Oral epithelial cells from drinking containers.* The swab was moistened by squeezing three drops of molecular biology grade water (approximately 25–30 μl) onto the swab head. The rim of a ceramic mug, plastic container, bottle, styrofoam cup, or coffee lid was rubbed with the moistened swab while rotating the swab to maximize sample collection. Twenty oral epithelial cell samples from 18 unique individuals were processed in the fully integrated ANDE system.
- *Blood on FTA paper*. A total of 48 samples were processed using blood collected in sodium heparin anticoagulant. A set of 24 samples was collected from 19 unique donors and a second set of 24 samples from a single donor. Dried blood samples were prepared by pipetting 150 μl of fresh whole blood onto FTA paper (Whatman Cat#WB120211) and allowed to dry at room temperature for at least overnight. For each sample, three 3-mm discs were punched and incubated for 15 min at 50 °C in TE^-4^ buffer (10 mM Tris–HCl pH 8, 0.1 mM EDTA). The TE^-4^ solution was collected on a swab and inserted into a LDC BioChipSet for automated processing.
- *Blood on untreated paper*. A total of 48 samples were processed. A set of 24 samples was collected from 20 unique donors and a second set of 24 samples from a single donor. Three 3-mm discs from each sample were prepared as above using untreated paper (Whatman 903™ Protein Card Saver Ref#10534612).
- *Buccal cells on FTA* paper. A total of 48 samples were processed. A set of 24 samples was collected from 24 unique donors, and a second set of 24 samples from a single donor buccal samples were collected using the Easicollect™ Buccal Collection Kit (Whatman™ Cat#WB120237) following the manufacturer’s protocol. Three 3-mm discs from each sample were prepared as above.
- *Buccal on untreated paper*. A total of 48 samples were processed. A set of 24 samples was collected from 24 unique donors, and a second set of 24 samples was collected from a single donor. Buccal samples were collected using Buccal DNA Collector (Bode Technology Cat#P01D28) following manufacturer’s protocol. Three 3-mm discs from each sample were prepared as above.
- *Dried blood on ceramic tile.*
  - ▪ Reproducibility study. Blood samples from 10 unique donors were processed. The samples were collected by fingerstick, and one 5 μl, two 3 μl, and one 1 μl samples were spotted onto a ceramic tile from each donor; a total of 40 samples. The blood was allowed to dry overnight at room temperature.
  - ▪ Accuracy/concordance study. Ten samples from ten unique individuals were processed. Fingerstick samples of 5 μl were spotted onto clean ceramic tiles and allowed to dry overnight at room temperature.
  - ▪ Sensitivity study. Seven blood samples from two unique individuals were processed. From each donor, fingerstick samples of 25 μl, one 10 μl, one 5 μl, two 3 μl, one 1 μl, one 0.5 μl, and one 0.1 μl were spotted onto a ceramic tile. Samples containing less than 1 μl were prepared by dilution with 1× phosphate-buffered saline. Samples were allowed to dry at room temperature overnight or longer. Using a moistened swab, the dried blood was collected as described above.
- *Dried blood on clothing.* Fresh whole blood was collected and stored in an anticoagulant-containing vacutainer tube, and approximately 100 μl was transferred onto cotton and denim fabrics. The swab was pre-moistened with sterile water and used to swab the bloodstain. Swabs were inserted into biochipsets for processing.
- *Dried semen on clothing.* Tubes containing semen samples were gently mixed, and approximately 100 μl of each sample was transferred onto cotton or denim fabric. The semen was allowed to dry at least overnight at room temperature. The swab was pre-moistened with sterile water and used to swab the sample. Next, 50 μl of freshly prepared 150 mM DTT was loaded while rotating the swab to ensure coverage of the entire swab. Swabs were inserted into biochipsets for processing.
- *Chewing gum.* The swab was moistened with sterile water in a drop dispenser bottle by squeezing three drops onto the swab head. With the moistened swab head, the entire exterior of the gum was thoroughly swabbed. Swabs were inserted into biochipsets for processing.
- *Cigarette butt.* Each cigarette butt was cut in half, and the filter paper was separated from the filter by cutting the paper lengthwise with the scalpel and peeling the paper off with sterile disposable forceps. With the exterior side of the filter paper facing up, the paper was cut in half lengthwise. One of the filter paper halves was cut into two pieces, and each portion was again cut into four smaller pieces, for a total of eight pieces (each piece measured approximately 1/8″ × 1/4″). Using sterile disposable forceps, each filter piece was picked up and carefully dropped into the swab chamber, ensuring the pieces fall into the bottom of the chamber. A standard NetBio swab cap was inserted into the BCS to seal the swab chamber prior to analysis.
- *Cellphone.* The swab was moistened with sterile water in a drop dispenser bottle by squeezing three drops onto the swab head. With the moistened swab head, the entire surface of the cellphone was thoroughly swabbed with focus on the screen. The swab head was rotated to maximize collection of any biological material deposited.
- *Bone.* Bone fragments (postmortem index of <8 h to 6 days) measuring ½″ around from the femur shaft were prepared by milling, and 10 mg bone powder was collected and transferred into a sterile 2-ml microfuge tube. To the tube, 120 μl of demineralization buffer was added and vortexed for 1 min. The undissolved bone particulates were separated from the demineralized solution by centrifugation for 2 min at 20,000 *g*. The liquid was then pipetted and loaded onto a NetBio swab for rapid DNA processing.

### Sample processing on ANDE system

System operation was performed as previously described [30]. Briefly, the operator is prompted to scan each swab sample’s cap using the on-board RFID reader and to insert the sample into the BCS. After the fourth swab has been loaded, the touchscreen prompts the operator to insert the LDC BCSC into the instrument and close the door to commence sample processing. The fully integrated LDC run is completed in 102 min. The Powerplex 16 HS assay was used, and accuracy and concordance samples were submitted to Cellmark Forensics LabCorp Specialty Testing Group (Dallas, TX) for conventional processing.

### Expert System

The ANDE Expert System Software processes the raw data, assigns allele designations, and employs rules to interpret the DNA profiles. The Expert System software was specifically designed and developed for the analysis of ANDE data and is fully integrated with no user intervention required. Immediately following an ANDE run, optical data generated during electrophoresis is subjected to signal processing, which includes setting the baseline to zero and performing color correction. The Expert System then evaluates the internal lane standard and the allelic ladder using a strict set of criteria. Then, a series of rules are fired to assign alleles and evaluate locus- and sample-specific criteria such as peak height, stutter, and heterozygote peak height ratio. At the conclusion of the evaluation, the ANDE Expert System generates the following outputs:

- Allele table listing all passing allele calls for all samples
- .png file (electropherogram) for rapid output visualization
- .xml file for upload to combined DNA index system (CODIS)
- .fsa file to permit review with conventional software packages

## Results and discussion

### Sample concentration by integrated microfluidic ultrafiltration

The HDC biochipset was initially designed for the purification and analysis of DNA prepared from buccal swabs. A typical buccal swab contains in excess of 1 μg of genomic DNA, but less than one thousandth of this amount is required to generate an STR profile. Accordingly, the HDC BioChipSet purification module was designed to reduce recovered DNA at essentially every process step. In contrast, touch samples will often contain much less than 10 ng of DNA, so it was critical to modify the purification module to optimize DNA purification efficiency. Accordingly, three fundamental changes were made to the HDC biochipset: (1) the percentage of cellular lysate subjected to DNA purification was increased to essentially 100 % of the cell lysate volume), (2) the efficiency of DNA binding to the purification filter was increased, and (3) the purified DNA was concentrated prior to amplification.

The DNA concentration membrane was chosen to allow retention of all DNA molecules, meet desired flow rates, and exhibit chemical compatibility with biochipset fabrication processes. Filtration stops when the DNA solution fills the concentration chamber. By design, the volume of the concentrated solution is approximately 25–30 μl. The concentrated DNA is then transferred pneumatically from the ultrafiltration chamber for subsequent PCR amplification. To assess the performance of the sample concentration module, 250-μl solutions containing varying quantities of purified human genomic DNA were passed through the concentration module. At 50, 5, and 1 ng of input DNA, 82, 84, and, 99 % of the DNA was recovered, respectively, indicating that the module functions appropriately across the range of DNA expected to be found in low DNA content samples. An average volume concentration of 9.6-fold was observed in these experiments. Incorporating the sample concentration module brings the total automated script time to 102 min.

### Reproducibility

#### Blood and buccal samples on FTA and untreated paper

**Fig. 3:**
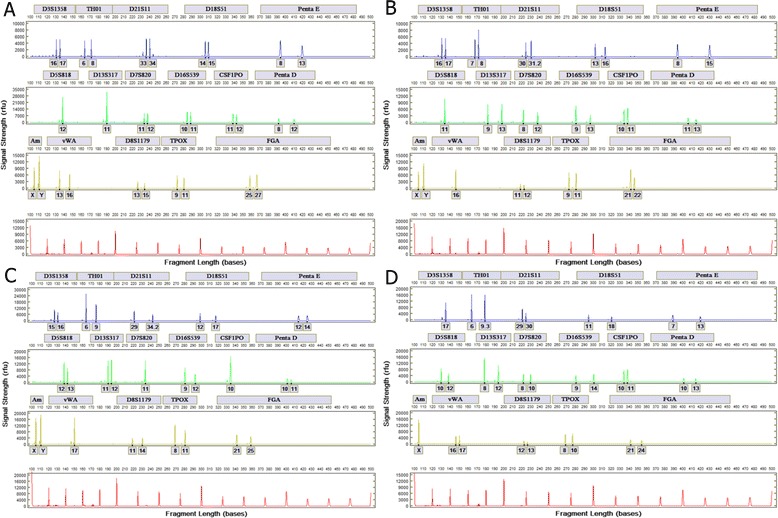
STR profiles from automated processing of reference samples. **a** Blood on FTA paper. **b** Blood on untreated paper. **c** Buccal cells on FTA paper. **d** Buccal cells on untreated paper

System reproducibility was assessed with 24 blood samples on FTA paper and untreated paper and 24 buccal cell samples on FTA paper and untreated paper. Each set of 24 samples collected from one unique donor. Figure 3 shows representative profiles from the four sample types. Table 1 displays the results of the reproducibility study from the blood and buccal cells on FTA and untreated paper samples.

**Table 1:** Reproducibility of Blood and Buccal cells on FTA and untreated paper

		Experiment			
		Blood on FTA paper	Blood on untreated paper	Buccal cells on FTA paper	Buccal cells on untreated paper
Replicate	1	Y	Y	Y	Y
	2	Y	Y^*^	Y	Y
	3	Y	Y	Y	Y
	4	Y	Y	Y	Y
	5	Y	Y	Y	Y^*^
	6	Y	Y	Y	Y
	7	Y	Y	Y	Y
	8	Y	Y	Y	Y
	9	Y	Y	Y	Y
	10	Y	Y	Y	Y
	11	Y	Y	Y	Y
	12	Y	Y	Y	Y
	13	Y	Y	Y	Y
	14	Y	Y	Y	Y
	15	Y	Y	Y	Y
	16	Y	Y	Y	Y
	17	Y	Y	Y	Y
	18	Y	Y	Y	Y
	19	Y	Y	Y	Y
	20	Y	Y	Y	Y
	21	Y^*^	Y	F	Y
	22	Y	Y	Y	Y
	23	Y	Y	Y	Y^*^
	24	Y	Y	Y	Y

*Y* a full STR profile was obtained with all alleles concordant; 91 samples met this criterion. Y* a partial profile was generated with all alleles concordant; 4 samples met this criterion. *F* a sample did not generate a profile; 1 sample failed to generate a profile. In total, 95 of the 96 samples generated profiles, and all were concordant

#### Dried blood samples

**Fig. 4:**
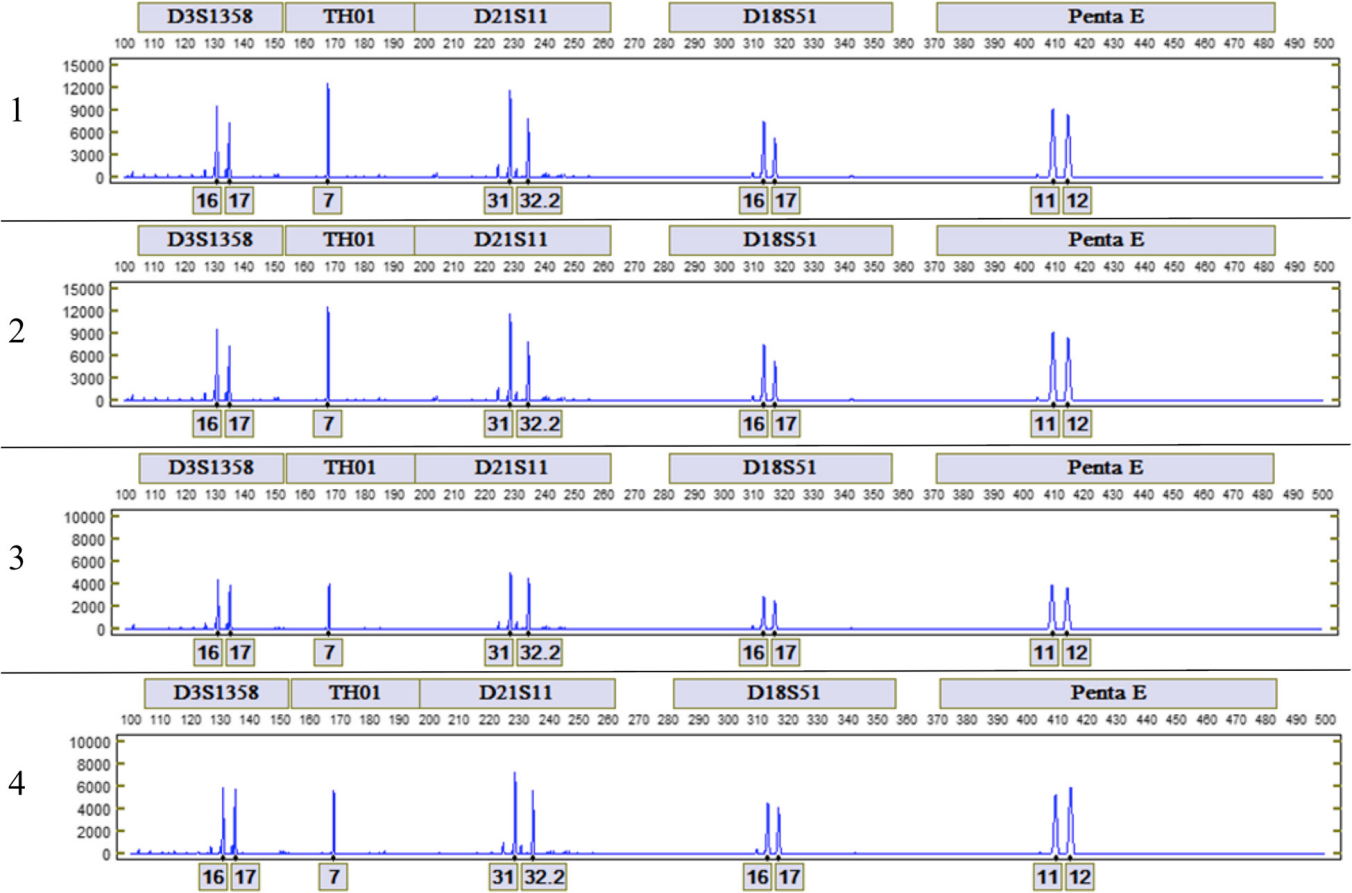
Comparison of the alleles in the Fluorescein (FL) dye channel for each of four replicates of a representative donor. From *top* to *bottom* are FL profiles from 5, 3, 3, and 1 μl dried blood samples

The consistency at which the LDC system generates DNA profiles from swab-in to result-out without user intervention was also evaluated by processing 5, 3, 3, and 1 μl dried blood samples from 10 unique donors. From the 40 samples processed, 30 generated full PP16 profiles, 8 generated profiles with 14 or 15 called loci, 1 sample (1 μl dried blood) had dropouts at TH01 and vWA due to low signal, and 1 sample did not generate a profile due to a microfluidic failure. A comparison of the automated Expert System calls in the fluorescein (FL) dye channel for each of the four replicates of a representative donor is shown in Fig. 4.

**Fig. 5:**
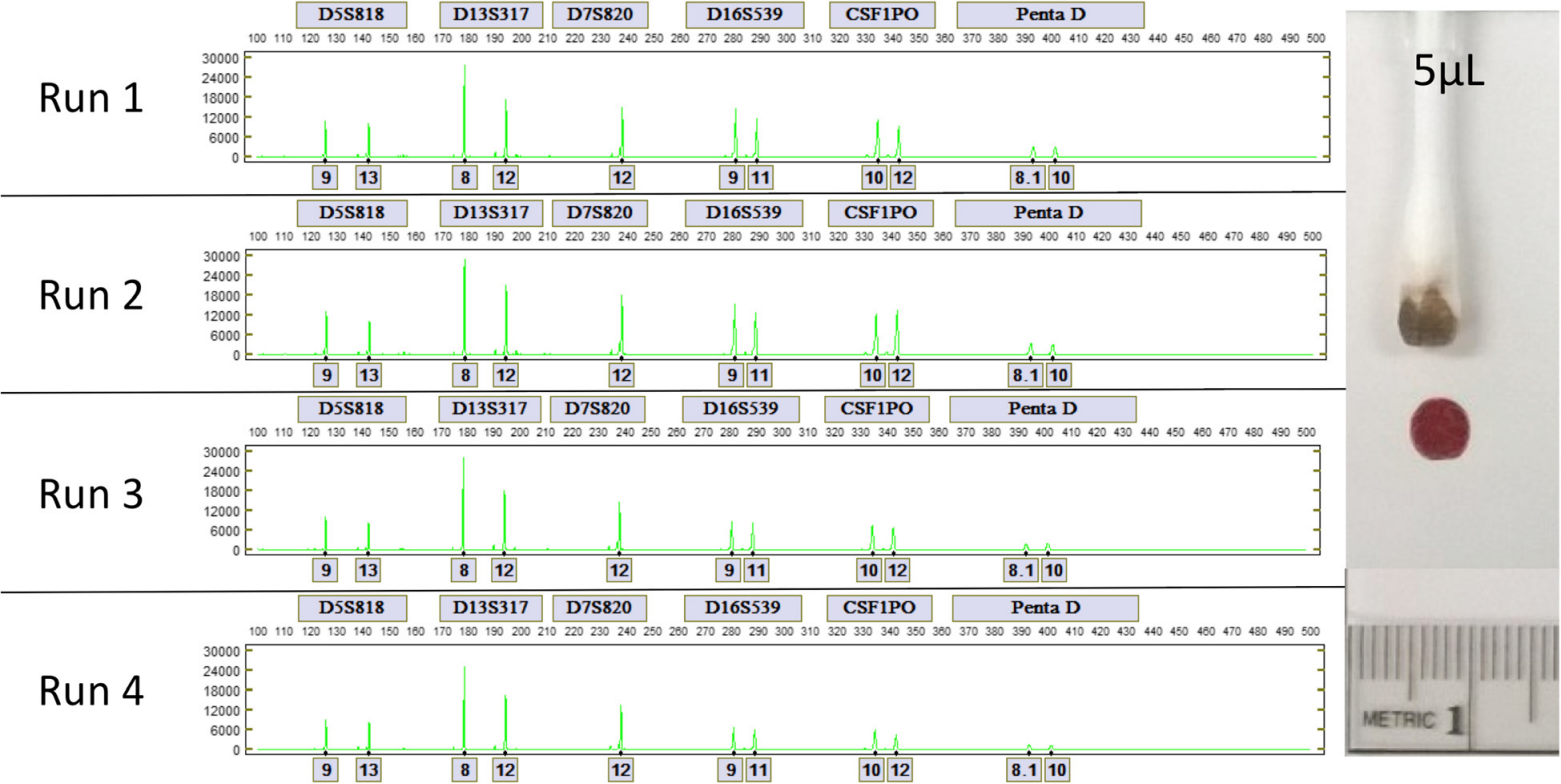
Comparison of the alleles in the JOE dye channel for each of four 5 μl dried blood samples. A 5 μl dried blood sample is approximately 0.5 mm in diameter

Swabs were collected from 5 μl dried blood samples of one unique donor and analyzed in four independent LDC runs to evaluate inter-run reproducibility. As expected, all samples generated full PP16 profiles. A comparison of the automated calls in the JOE (green label) dye channel for each of the four run replicates is shown in Fig. 5.

### Accuracy/Concordance

The ability of the system to generate concordant allele calls was assessed by processing a total of 262 samples from sample types including dried blood (50 samples from 20 unique donors), oral epithelial cells from drinking containers (20 samples from 18 unique donors), blood on FTA paper (24 samples from 19 unique donors and 24 samples from a single donor), blood on untreated paper (24 samples from 20 unique donors and 24 samples from a single donor), buccal cells on FTA paper (24 samples from 24 unique donors and 24 samples from a single donor), and buccal cells on untreated paper (24 samples from 24 unique donors and 24 samples from a single donor). Total alleles assessed for concordance in this study is 8094, with 8091 (99.963 %) concordant and 3 (0.036 %) discordant. Three dropouts were observed, two in the 1-μl dried blood sample noted above and one from a Styrofoam mug. Low DNA content samples are at higher risk for stochastic events leading to dropouts and dropins as compared to high DNA content samples.

### Sensitivity

**Fig. 6:**
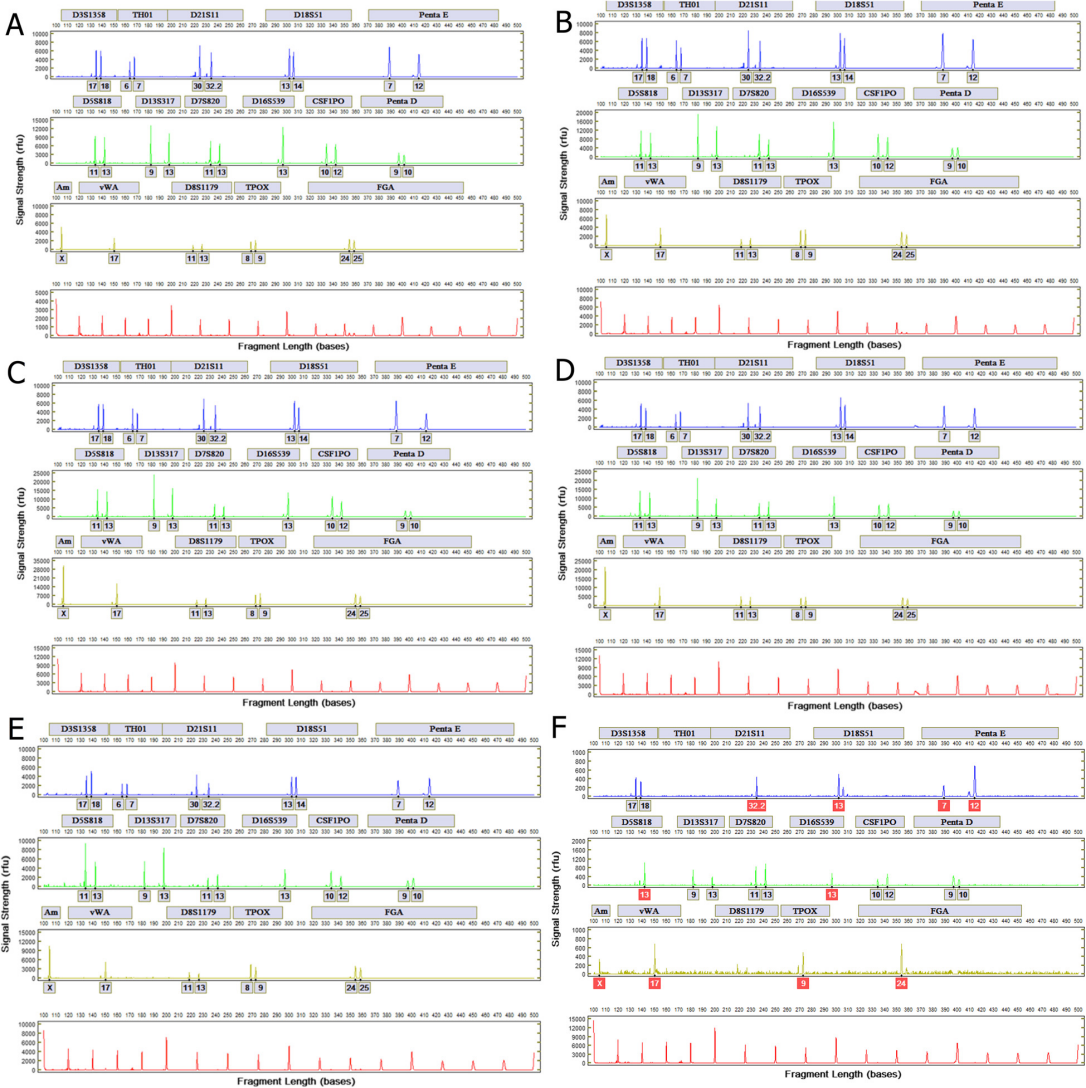
Representative profiles from dried finger prick blood samples **a** 25 μl, **b** 10 μl, **c** 5 μl, **d** 3 μl, **e** 1 μl, and **f** 0.1 μl. The automated Expert System reports peaks in *red warning boxes* if analytical threshold of signal strength and peak-height ratio are not met. Peaks indicated in *red warning boxes* are not included in the automatically-generated .xml file

Full profiles were generated from samples with 1 μl and more of blood. Partial profiles with less than 10 CODIS loci are generated when less than 1 μl is used. Figure 6 shows representative profiles generated from varying inputs of blood for analysis. At 0.1 μl (prepared as 10-fold dilution of 1 μl), 5 loci (D3S1358, D13S317, D7S820, CSF1PO, and Penta D) were called.

### Precision, resolution, and peak height ratio

#### Precision

**Fig. 7:**
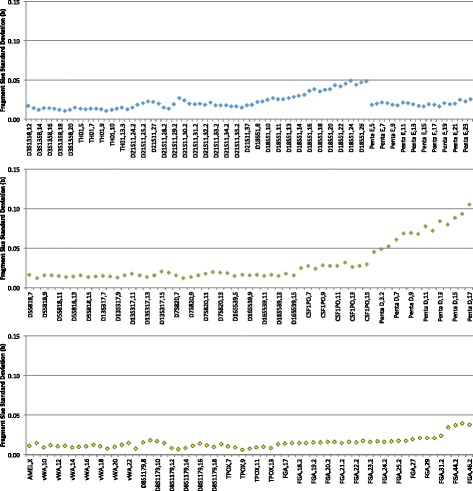
Inter-run precision of the system based on 75 runs on 7 ANDE instruments. Plots of allelic ladder fragment size standard deviation in bases versus locus/allele are presented for each fluorescent dye

Inter-run precision was assessed by evaluating 75 allelelic ladders across 7 instruments. Precision is expressed as the standard deviation of the fragment size difference (in bases) of each of the 210 allelic ladder fragments. Standard deviation ranges from 0.0070 for TPOX 9 to 0.1052 for Penta D 17 (Fig. 7). These standard deviations are well below the acceptable level of <0.16 bases, demonstrating that the system is capable of precise sizing and designating of alleles.

#### Resolution

System resolution was calculated for 386 samples generated during this study.

**Fig. 8:**
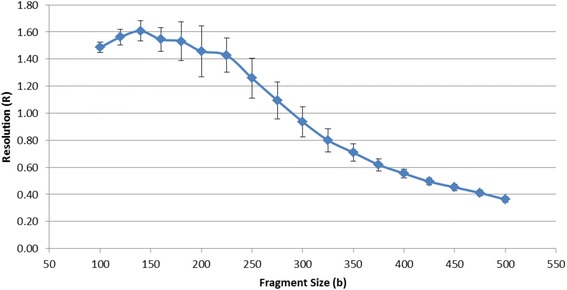
Resolution of the system as determined from 386 samples on 7 instruments. *R* was calculated as previously described [31]. *Error bars* represent 1 standard deviation

Figure 8 shows that the resolution ranges from 1.607 at 140 bases to 0.362 at 500 bases. *R* values are well above 0.2, demonstrating that the system is capable of single base resolution across the 100 to 500 base sizing range of the assay.

#### Peak height ratio

**Fig. 9:**
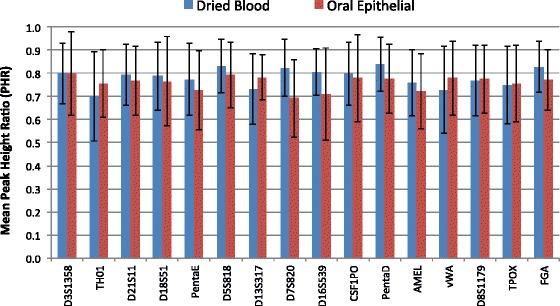
Peak height ratio (PHR) analysis of STR profiles from dried blood and oral epithelial cell samples. Data is compiled from 50 dried blood samples and 20 oral epithelial samples from drinking containers. *Error bars* represent 1 standard deviation

For dried blood and oral epithelial samples, peak height ratio (PHR) for each locus was calculated for each of the 70 samples. The PHR was calculated for heterozygous loci by taking the ratio of the signal strength of the weakest allele to the signal strength of the strongest allele. The PHR and standard deviation by locus for each sample type is shown in Fig. 9. The PHRs were typically 0.7–0.8 across loci, as expected for LDC samples.

### Rapid DNA processing of additional forensic sample types

**Fig. 10:**
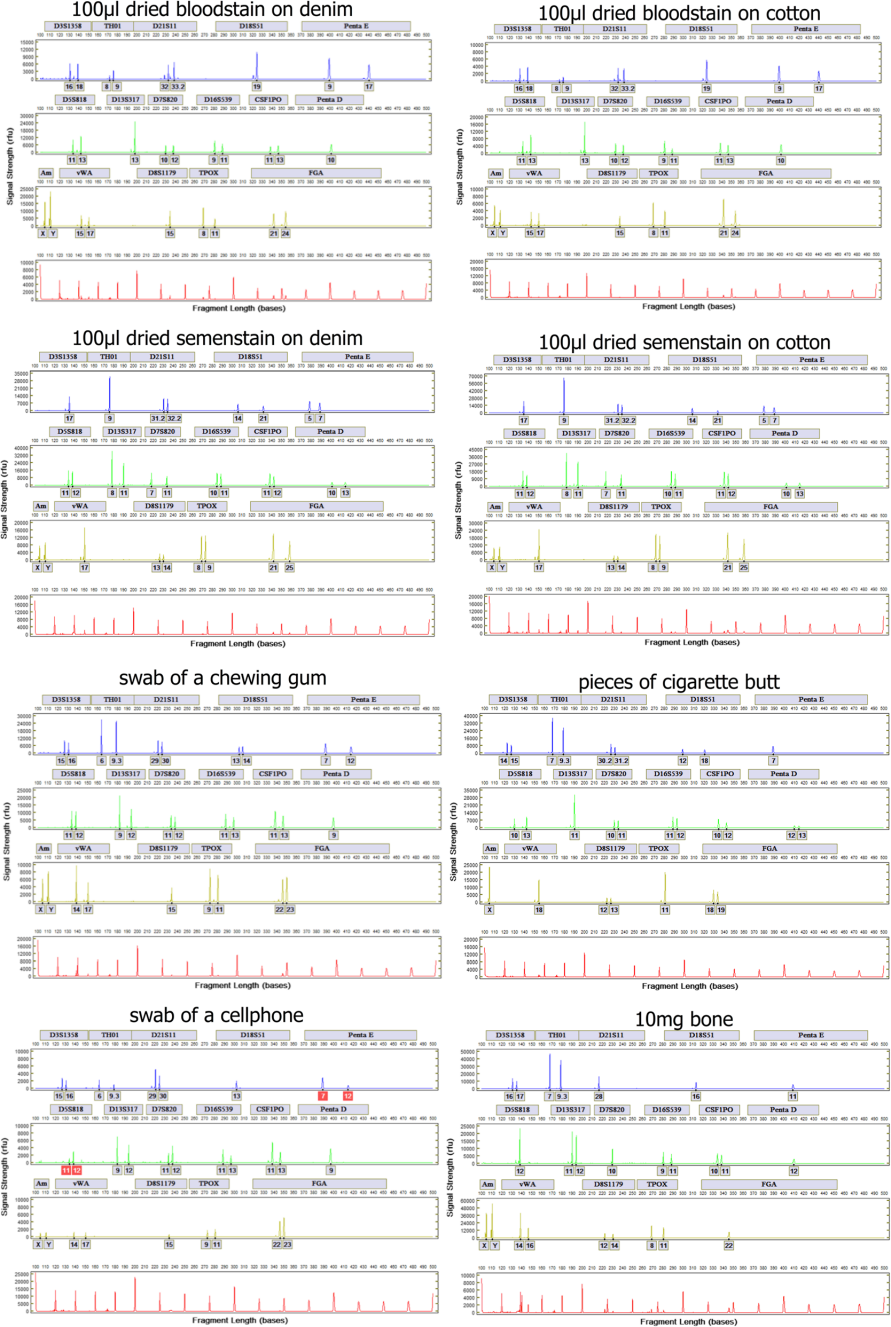
STR profiles from several forensic sample types. Generated profiles are concordant. In the swab of cellphone, alleles in PentaE and D5S818 are flagged in *red* by the Expert System due to low signal and PHR imbalance

The ability of the LDC system to process other forensically relevant sample types was also investigated. Profiles generated from dried bloodstain on clothing, dried semen on clothing, chewing gum, cigarette butt, cellphone, and bone using the LDC BioChipSet are presented in Fig. 10.

## Conclusions

The LDC content BioChipSet Cassette was been designed to accept and process samples containing relatively low quantities of DNA. Taken together, the data demonstrates that the ANDE instrument, LDC BioChipSet, and Expert System Software perform reliably with acceptable accuracy, concordance, precision, resolution, and PHR. The LDC BioChipSet generates STR profiles from a broad range of samples including blood spatter stains, blood and buccal samples on FTA and untreated paper, casework samples such as cigarette butts and oral epithelium of cups and glasses, and disaster samples including muscle and bone. The fully integrated Expert System is capable of interpreting a wide range or sample types and input DNA quantities, allowing samples to be processed and interpreted without a technical operator.

The ability to rapidly process this broad range of sample types has the potential to positively impact military, law enforcement, intelligence, and human trafficking investigations [32]. It is noted that the current system does not quantify human DNA prior to PCR amplification and, accordingly, the STR profiles generated from casework samples in NDIS and SDIS laboratories cannot be utilized to search the FBI’s criminal justice DNA databases. However, the data can be utilized to generate investigative leads and, in many jurisdictions, can be utilized to search national and local databases. In addition, data from an NDIS-approved system (pending for the ANDE system) can be utilized to search profiles from reference samples, including samples on FTA and untreated papers.

The rapid processing of bone and muscle (as well as teeth, liver, and other samples—data not shown) samples suggests a critical role for rapid DNA in disaster victim identification. In addition, an expanded STR locus set based on a previously published 26plex has been applied to the LDC system. The advantages of an expanded STR set in this context are that (1) degraded DNA samples are more easily identified with a larger number of smaller molecular weight loci and (2) kinship (based on non-obligatory alleles) is better established with a larger set of loci. As rapid DNA gradually becomes a routine tool in the forensic armamentarium, both in the lab and at filed-forward locations, its applications and utility are likely to expand dramatically.

## Abbreviations

ANDE: accelerated nuclear DNA equipment
BCS: BioChipSet
CODIS: combined DNA index system
LDC BCSC: low DNA content BioChipSet cassette
PCR: polymerase chain reaction
STR: short tandem repeats
UF: ultrafiltration
